# Incorporation of Levodopa into Biopolymer Coatings Based on Carboxylated Carbon Nanotubes for pH-Dependent Sustained Release Drug Delivery

**DOI:** 10.3390/nano8060389

**Published:** 2018-05-31

**Authors:** Julia Meihua Tan, Bullo Saifullah, Aminu Umar Kura, Sharida Fakurazi, Mohd Zobir Hussein

**Affiliations:** 1 Materials Synthesis and Characterization Laboratory, Institute of Advanced Technology (ITMA), Universiti Putra Malaysia, Serdang 43400, Selangor, Malaysia; julia.tanmh@gmail.com (J.M.T.); bullosaif1@gmail.com (B.S.); 2 Laboratory of Vaccine and Immunotherapeutics, Institute of Bioscience (IBS), Universiti Putra Malaysia, Serdang 43400, Selangor, Malaysia; aminuukura@yahoo.com (A.U.K.); sharida.fakurazi@gmail.com (S.F.); 3 Department of Human Anatomy, Faculty of Medicine and Health Sciences, Universiti Putra Malaysia, Serdang 43400, Selangor, Malaysia

**Keywords:** single wall carbon nanotubes, nanomedicine, Parkinson’s disease, 3T3, MTT assay, biopolymers

## Abstract

Four drug delivery systems were formulated by non-covalent functionalization of carboxylated single walled carbon nanotubes using biocompatible polymers as coating agent (i.e., Tween 20, Tween 80, chitosan or polyethylene glycol) for the delivery of levodopa, a drug used in Parkinson’s disease. The chemical interaction between the coating agent and carbon nanotubes-levodopa conjugate was confirmed by Fourier transform infrared (FTIR) and Raman studies. The drug release profiles were revealed to be dependent upon the type of applied coating material and this could be further adjusted to a desired rate to meet different biomedical conditions. In vitro drug release experiments measured using UV-Vis spectrometry demonstrated that the coated conjugates yielded a more prolonged and sustained release pattern compared to the uncoated conjugate. Cytotoxicity of the formulated conjugates was studied by 3-(4,5-dimethylthiazol-2-yl)-2,5-diphenyltetrazolium bromide (MTT) assay using normal mouse embryonic fibroblast 3T3 cell line. Compared to the non-coated conjugate, the MTT data indicated that the coating procedure improved the biocompatibility of all systems by 34–41% when the concentration used exceeded 100 μg/mL. In conclusion, the comprehensive results of this study suggest that carbon nanotubes-based drug carrier coated with a suitable biomaterial may possibly be a potential nanoparticle system that could facilitate drug delivery to the brain with tunable physicochemical properties.

## 1. Introduction

Parkinson’s disease is a chronic degenerative disorder of the central nervous system that commonly affects one in every 100 adults above 65 years of age [1]. The disease is characterized by low level of dopamine in the brain and eventually may lead to severe difficulties with body motions such as rigidity, tremor, unstable posture and slowness of body movements. To manage the disease symptoms, patients are treated with levodopa (LD). Orally administered LD is absorbed and converted into dopamine in the brain through the blood–brain barrier (BBB) [2]. However, once administered, LD is actively metabolized in the periphery as well as in the central nervous system and, only a small amount reaches the brain. Therefore, to achieve maximum therapeutic efficacy, LD is taken in combination with a peripheral decarboxylase inhibitor called carbidopa. This is to prevent LD from being broken down rapidly in the peripheral system before it reaches the brain [3]. Apart from that, patients receiving long-term LD therapy generally experience a “wearing off” effect which in turn shortens the duration of benefit from each dose. Hence, as the treatment progresses, more frequent LD administration is necessary [4]. This often results in adverse effects such as restlessness, nausea, low blood pressure and muscle pain. As such, researchers are actively looking into various nanotechnological methods which could possibly increase LD’s bioavailability in the central nervous system with improved drug efficacy. The main strategies currently being developed are drug delivery systems [5], efflux pump mechanisms [6], prodrugs [7], lipophilic analogs, intraventricular drug infusion, hyperosmotic opening of the BBB and the bypass of the BBB through intracerebral delivery [8,9]. In the field of drug delivery applications, nanoparticles (carbon nanotubes, dendrimers, liposomes, micelles, etc.) are the most popular studied nanoscale materials for transportation of different therapeutic agents directly into the targeted site of action [10].

Among various nanoparticles described above, functionalized carbon nanotubes (CNT) have evolved as the enthusiastically researched therapeutic carrier for targeted drug delivery to the central nervous system after the recent discovery of their ability to penetrate cellular membranes [11]. Despite that, transporting molecular drugs into brain is still a crucial challenge and is greatly restricted due to the existence of the BBB [12]. The BBB is a highly selective semipermeable complex structure formed by the brain endothelial cells, thereby constituting a physiological barrier that tightly regulates the passage of ions, molecules and cells between the blood and the brain. Nevertheless, many successful studies have been reported in the literature regarding the uptake of chemically functionalized CNT into the brain by different kinds of neural tissue cells such as microglia, neurons and astrocytes [13,14,15]. In addition, a recent transformative breakthrough of CNT is in the application of neural interfaces owing to their electrically conductive properties which arise from the conjugated π–electron systems [16]. Structurally, the nanotubes not only possess anisotropic conductivity features which mimic to those of neurons but also having dimension parallel to that of dendrites (the branched protuberance from neurons). Motivated by this fact, a group of scientists conducted in vivo chronic studies in Parkinsonian rodents and they discovered that CNT fiber microelectrodes are capable in stimulating mouse neurons as effectively as metal electrodes while producing lower levels of inflammation [17]. This implies that electrical stimulation of the nervous system can be used to treat symptomatic conditions related to Parkinson’s disease and therefore, could further help patients recover muscular and sensory functions.

In this elementary work, carboxylated single walled carbon nanotubes (SWCNT) were chosen as the nanoparticle-mediated delivery carrier due to their intrinsic nanostructure (length-to-diameter ratio of up to 132,000,000:1), high cargo loading capacity, ultralight weight, non-immunogenic property and good chemical stability [18]. As a nanoscale platform for drug delivery, their external surfaces can be chemically functionalized with desired bioactive peptides [19], genes [20] or drugs [21], while their internal cavities can be encapsulated with bioactive molecules [22]. Furthermore, these nanomaterials are capable of transporting and translocating therapeutic molecules directly into the targeted cell cytoplasm through nanoneedle-like mechanism without inducing cellular apoptosis [23]. However, despite all the potential advantages highlighted above, pure nanotubes are highly hydrophobic in nature due to the van der Waals forces and tend to form aggregates which may cause characteristic cell changes and apoptosis when administered into human body. To overcome this technical barrier, scientists used to attach hydrophilic functional groups such as hexadecyltrimethylammonium bromide, sodium dodecyl sulfate [24], sodium cholate [25] onto the hydrophobic surfaces of SWCNT as an attempt to impart water-solubility and biocompatibility to the nanotubes. Even though chemically modified CNT have proven to enhance dispersibility in aqueous medium, there are findings suggested that surface functionalization of carboxyl and hydroxyl groups may contribute to a higher extent of in vitro cytotoxicity as compared to non-functionalized ones [26]. To further avoid the cytotoxicity possibly induced by the functionalized SWCNT, we aimed to incorporate LD into four different biopolymer coatings, namely Tween 20, Tween 80, chitosan and polyethylene glycol, based on carboxylated SWCNT as an effort to improve the delivery of LD in a more sustained and effective manner. These coating agents are reported to efficiently disperse bundled nanotubes into aqueous suspension of individual nanotubes by stabilizing the effect of their hydrophobic surfaces.

**Scheme 1:**
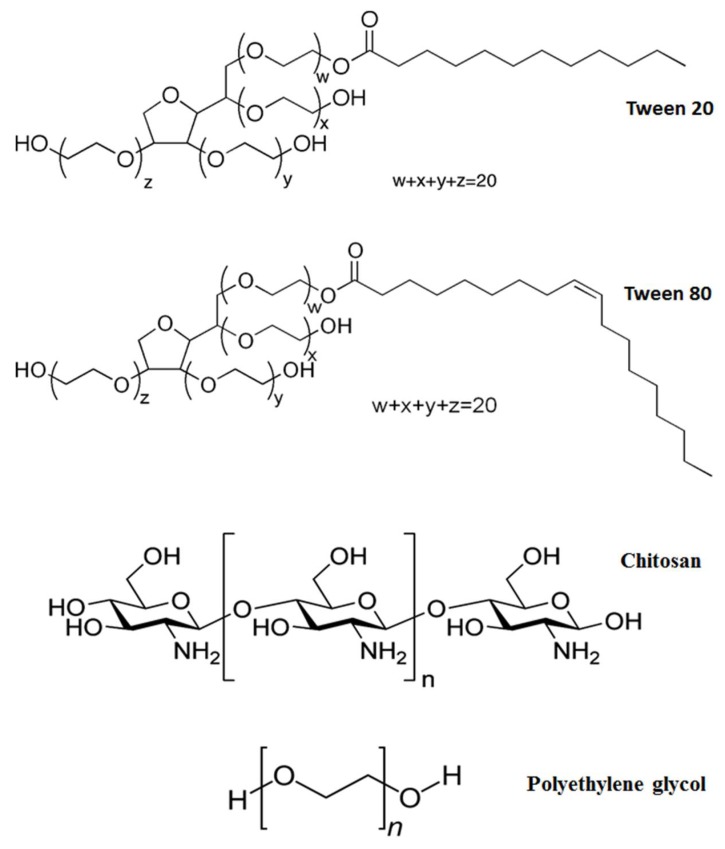
The chemical structures for Tween 20, Tween 80, chitosan and polyethylene glycol.

Tween 20 (T2) and Tween 80 (T8), as non-ionic polyoxyethylene surfactants, have been widely used in the preparation of pharmaceutical and consumable products for both preventing non-specific surface adsorption and as stabilizers against protein aggregation [27,28]. In fact, nanoparticles that are coated with Tweens were found to be able to specifically transport several drugs such as doxorubicin [29], loperamide [30], tacrine [31], tubocurarine [32] and hexapeptide dalargin [33] to the brain after administration by enhancing the permeability of the BBB. These Tween series can be distinguished by the content of fatty acid esters with the monooleate fraction of T8 making up to >58% and the monolaurate fraction of T2 making up to approximately 40–60% of the mixture [34]. The chemical structures of the Tween molecules are shown in Scheme 1.

Chitosan (CS), a linear polysaccharide obtained by alkaline deacetylation of crustacean chitin, has been prominently used for surface coating of CNT for various drug delivery systems [35] due to its biodegradable, biocompatible, non-toxic and antimicrobial property. This abundantly available biopolymer can impart aqueous solubility and, thus, improve biological compatibility of CNT for drug delivery applications. In addition, it can be wrapped around CNT through electrostatic interactions and hydrophobic forces without a linker [36]. Polyethylene glycol (PG), on the other hand, is considered as a versatile coating material in the field of nanoparticle-assisted drug delivery system [37]. This non-ionic material with a monomer unit of –O(CH_2_)_2_, is generally known to exhibit very low toxicity and, hence, it is widely used as part of the pharmaceutical formulations in suppository, water-soluble ointment and pill. Due to its polar (from oxygen atom) and non-polar (from (CH_2_)_2_) groups, it has also been commonly used as a vehicle for the loading of hydrophobic drug molecules to improve their dissolution characteristics or aqueous solubility [38]. Furthermore, the circulation time of the PG-coated drug delivery system in the bloodstream can be extended from approximately 1 h to 1–2 days [39]. The chemical structures of CS and PG are demonstrated in Scheme 1.

For this study, we prepared and investigated the drug delivery profiles of LD-loaded carboxylated SWCNT (SWLD) in the presence of four different biopolymer coatings. The prepared samples were denoted as T2-SWLD (SWLD coated with Tween 20), T8-SWLD (SWLD coated with Tween 80), CS-SWLD (SWLD coated with chitosan) and PG-SWLD (SWLD coated with polyethylene glycol). We then evaluated the cytotoxicity effect of these coating agents on standard mouse embryonic fibroblasts 3T3 cell line because of their capacity for self-renewal, tissue remodeling and repair [40]. At present, there are no experimental studies concerning the cytotoxicity of SWCNT, especially on drug-conjugated CNT with the extensively used T2, T8, CS and PG coating agents on normal standard fibroblasts. It is critically important to evaluate the toxicity of CNT conjugates since these coating materials are frequently being used in research laboratories and manufacturing industries.

## 2. Materials and Methods

Commercial SWCNT functionalized with carboxyl group (–COOH) were used as the drug delivery platform. The nanotubes of 90% purity were purchased from Chengdu Organic Chemicals Co. Ltd., Chinese Academy of Sciences (Chengdu, China) and used without further purification. Their main characteristics are given in Table 1. Pure commercial LD (C_9_H_11_NO_4_, molecular weight 197.19) of 99% purity and PG (average molecular weight 300) were obtained from Acros Organics (Geel, Belgium). T2 (C_58_H_114_O_26_, polyoxyethylenesorbitan monolaurate), T8 (C_64_H_124_O_26_, polyoxyethylenesorbitan monooleate), CS (low molecular weight chitosan with 75–85% degree of deacetylation), phosphate buffered saline (PBS) solution and MTT (3-(4,5-dimethylthiazol-2-yl)-2,5-diphenyltetrazolium bromide) powder were obtained from Sigma-Aldrich (Saint Louis, MO, USA). PBS solution was prepared using PBS buffer tablet (pH 7.4) dissolved in 1 L of deionized water. Aqueous acetic acid solution of 99.8% purity was obtained from HmbG Chemicals (Hamburg, Germany) and was used as solvent for CS. Penicillin-streptomycin antibiotic, fetal bovine serum (FBS) and trypsin-EDTA were purchased from PAA Laboratories (Pasching, Austria). RPMI 1640 with L-glutamine was purchased from Nacalai Tesque Inc (Kyoto, Japan). Healthy fibroblast cell line (3T3) derived from mouse embryonic fibroblasts were supplied by the American Type Culture Collection (ATCC, Manassas, VA, USA). Analytical grade preparations were used for all the solvents and buffer solutions. Mili-Q plus System (Millipore, Burlington, MA, USA) was used for the preparation of deionized water and used in all experiments unless specified otherwise.

### 2.1. Instruments and Measurements

Fourier transform infrared (FTIR) analysis was used for characterization of functional groups using a Thermo Nicolet Nexus 671 spectrophotometer (model Smart Orbit) employing the KBr disc method, except for T2 and T8 by a direct deposition approach. Infrared spectra of the samples were measured in the range from 500 to 4000 cm^−1^ with 32 scans at a resolution of 2 cm^−1^. To estimate the degree of functionalization and imperfection of the samples, the intensity ratio of the D to G modes (I_D_/I_G_), Raman spectroscopy measurements were performed using a UHTs 300 Raman spectrometer (WITec, Germany) at a wavelength of 532 nm. To study the surface morphology of the samples, field emission scanning electronic microscopy (FESEM) was conducted using a Nova NanoSEM 230 microscope (FEI, Hillsboro, OR, USA) equipped with a field emission gun. Acceleration voltages applied were 10 kV at magnification of 200,000×. UV-Visible Lambda 35 spectroscopy (Perkin Elmer, Waltham, MA, USA) was used for LD concentration measurement for drug loading and drug release experiments. The polymer content of the coated SWCNT samples (without LD conjugation) namely T2-SWCNT, T8-SWCNT, CS-SWCNT and PG-SWCNT was investigated by thermogravimetric analysis (TGA) using a TA Instruments model Q500 (New Castle, DE, USA). The coating percentage of each biopolymer sample, T2-SWCNT, T8-SWCNT, CS-SWCNT and PG-SWCNT was found to be approximately 19%, 56%, 16% and 5%, respectively.

### 2.2. Preparation of Drug Loading onto Carbon Nanotubes

The loading of LD onto SWCNT functionalized with carboxylic acid moiety was conducted following previous method with some slight adjustment [41]. Briefly, 50 mg LD was dissolved in 400 mL of deionized water at the optimized drug concentration of 0.125 mg/mL. Approximately 400 mg nanotubes were dispersed into the drug solution and sonicated in a water bath for 30 min at room temperature. This was followed by continuous magnetic stirring for 24 h in the dark to maintain drug activity. The suspension was slowly adjusted to pH 4.0 for optimum absorption of LD onto nanotubes. Subsequently, the suspension was then centrifuged for 10 min at 4000 rpm, filtrated and rinsed with deionized water (3 cycles) and oven dried at 60 °C. The solid product (SWLD) was powdered and kept in a sample bottle for further use while the supernatant residue containing unbound LD was collected for the determination of drug loading capacity of SWCNT.

The amount of unbound LD in the supernatant was measured by a UV-Vis spectrophotometer at 280 nm (the characteristic absorbance wavelength of LD) with respect to the calibration curve accomplished under the same conditions. The results were then used to estimate the drug loading capacity according to the following equation:

Drug loading capacity (%) = (W_initial LD_ − W_unbound LD_)/W_initial LD_ (1)

where W is the weight in mg, and W_initial LD_ and W_unbound LD_ are the initial amount of LD and the amount of unbound LD in the supernatant residue, respectively. Using the equation, the LD loading capacity was calculated to be about 38%.

### 2.3. Preparation of Biopolymer Wrapping onto Drug-Loaded Carbon Nanotubes

Four different types of biopolymer, namely T2, T8, PG and CS were employed to enhance the biocompatibility level of SWLD through non-covalent surface wrapping. Briefly, SWLD (100 mg) were added into deionized water (100 mL) containing 1% T2, T8, PG or 0.5% CS (*v*/*v*) and magnetically stirred at room temperature for 24 h. After that, the biopolymer-wrapped SWLD was centrifuged and rinsed thoroughly with deionized water to remove any unbound polymer. Finally, the solid product was left to dry completely in an oven at 60 °C. The resulting products were named according to the corresponding biopolymer as T2-SWLD, T8-SWLD, PG-SWLD and CS-SWLD.

### 2.4. In Vitro Drug Release of LD

To study the drug release profile at 37 °C, 1 mg of sample was added into 3.5 mL of PBS solution at two different pH levels, i.e., pH 7.4 (to represent physiological environment) and 4.8 (to mimic human stomach after food intake). The experiment was then terminated upon reaching saturation and the accumulated release amount of LD in the medium was analyzed using UV-Vis spectroscopy at preset time intervals measured at 280 nm. The drug release results were fitted to five mathematical kinetic equations, i.e., zeroth order, first order, second order, Higuchi and Korsmeyer–Peppas models to study the release mechanism of LD from different types of biopolymer wrapped nanotubes.

### 2.5. Cell Culture

The 3T3 normal cell lines were cultured in a T25 culture flask in RPMI 1640 medium supplemented with 10% FBS and 1% penicillin/streptomycin (100 units/mL penicillin and 100 μg/mL streptomycin) in a humidified atmosphere at 37 °C in which the CO_2_ level was maintained at 5%. When reached 80% confluence, cells were subcultured in a new culture flask for seeding and treatment purpose using 0.25% trypsin-EDTA solution. To measure cell viability, cultured cells were seeded at a density of 1 × 10^4^ cells per well in 100 μL of culture medium into 96 well plate and incubated for 24 h to allow cell attachment. After that, the medium was replaced with fresh mediums containing T2-SWLD, T8-SWLD, CS-SWLD, PG-SWLD, SWLD and LD at various concentrations, and the treated cells were incubated for 72 h at 37 °C.

### 2.6. Cell Viability Assay

The in vitro cell cytotoxicity was assessed by the MTT assay through addition of 20 μL solution of freshly prepared MTT reagent (5 mg/mL in PBS) and incubation for 3 h at 37 °C until a purple colored formazan product developed. Then, the medium was discarded and replaced by 100 μL of dimethyl sulfoxide to dissolve the formazan. The absorbance was read on an absorbance reader model EL 800X (Winooski, VT, USA) after 1 h of incubation at a wavelength of 570 nm. All assays were done with three parallel samples in triplicate independently. The cytotoxicity of the samples was calculated as the percentage of cell viability with respect to control cells using the following equation:

Cell viability (%) = Abs_sample_/Abs_control_ × 100 (2)

where Abs_sample_ refers to the absorbance of the treated cells and Abs_control_ indicates the absorbance of the untreated cells.

### 2.7. Statistical Analysis

The obtained results were performed using the Statistical Package for Social Science (SPSS) version 22.0 software (Armonk, NY, USA) and data were expressed as mean ± standard deviation of triplicate. The results were analyzed by one-way analysis of variance (ANOVA). A probability of *p* < 0.05 was considered statistically significant.

## 3. Results

### 3.1. Characterization

**Figure 1:**
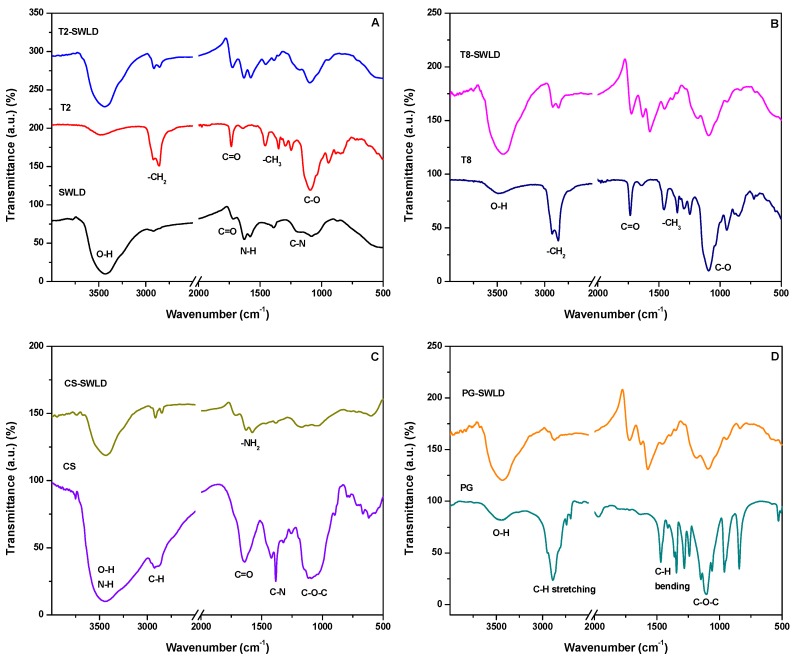
FTIR spectra of: SWLD, T2, T2-SWLD in (**A**); T8, T8-SWLD in (**B**); CS, CS-SWLD in (**C**) and PG, PG-SWLD in (**D**).

The FTIR spectra of SWLD, pure T2 and T2-SWLD are demonstrated in Figure 1a. Characteristic absorption peaks of T2 were observed at 3487, 2858, 1734, 1458 and 1092 cm^−1^, corresponding to the functional groups of –OH, –CH_2_, –C=O, –CH_3_ and –CO, respectively. Majority of the band positions of T2-SWLD are very similar to those of T2, with a slight shift to the lower wavenumber region, suggesting that significant chemical interaction between SWLD and T2 has taken place. Apart from that, the relative intensities of those bands were also detected in T8-SWLD, as shown in Figure 1b. This is because the molecular structure of T8 is quite similar to that of T2, except that T8 consists of mostly oleic acid. The FTIR characteristics of SWLD conjugate has already been extensively discussed in our previous work [41].

The FTIR spectrum of pure CS in Figure 1c showed that the broad absorption band corresponding to –OH and –NH stretching vibrations of alcohol and amine groups was observed at 3444 cm^−1^. The weak absorption band at 2925 cm^−1^ corresponds to the –CH stretching vibration of hydrocarbon while the band at 1640 cm^−1^ is attributed to the stretching of –C=O of acetamide group in CS. The absorption bands at 1420 and 1384 cm^−1^ are associated with the bending vibration of –CH and stretching vibration of –CN functional groups, respectively. In addition, the symmetric stretching vibration of C–O–C produced the broad peak at 1091 cm^−1^. For CS-wrapped SWLD, stretching vibrations from –OH, –NH, –CH and C–O–C were still observed at 3435, 2915, 2849 and 1169 cm^−1^, respectively, but they were slightly shifted to the right region. On the other hand, a new band was formed at 1577 cm^−1^ indicating the presence of –NH_2_ bending vibration from the pure CS molecules. 

The interaction of SWLD conjugate coated with PG is confirmed by FTIR spectra, as shown in Figure 1d. Intense absorption bands for PG-SWLD conjugate were observed at 3437, 2914, 2850, 1722, 1575 and 1097 cm^−1^. The FTIR band at 3437 cm^−1^ is attributed to –OH stretching vibration of the hydroxyl group in carboxylated nanotubes and PG molecules. The aliphatic –CH stretching was observed at 2914 and 2850 cm^−1^, while the –CH bending vibration was seen centered at 1575 cm^−1^. The absorption band at 1097 cm^−1^ shifted towards lower frequency compared to band at 1104 cm^−1^ for pure PG is due to the conjugation of C–O–C functional group with nanotubes. In addition, the absorption band due to –C=O stretching mode was seen located at 1722 cm^−1^ which is due to the carboxylic acid group in carboxylated nanotubes.

**Figure 2:**
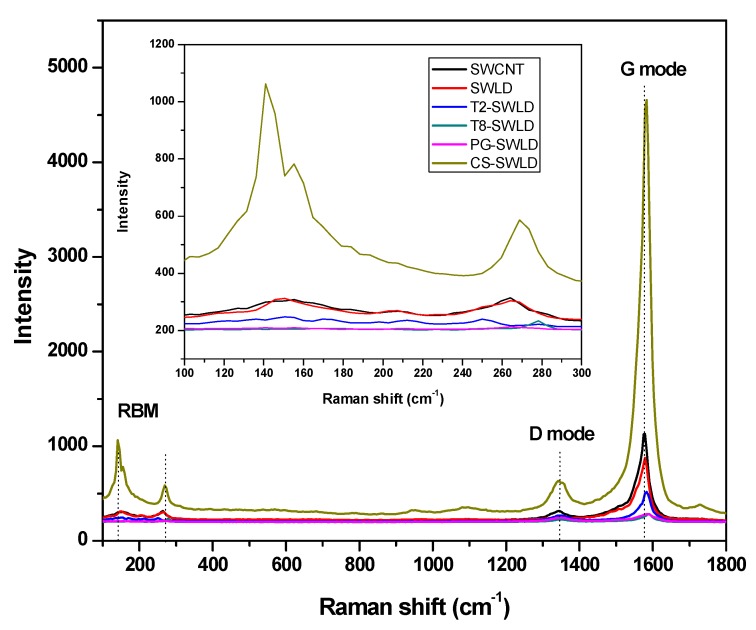
Raman spectra of carboxylated SWCNT, SWLD, T2-SWLD, T8-SWLD, PG-SWLD and CS-SWLD conjugates. Inset shows the Raman spectra of the RBM region for all the samples.

Based on Figure 2, the characteristic features of the graphitic layers in SWCNT were found in the region of 1346 cm^−1^ (disorder-induced D mode) and 1579 cm^−1^ (tangential G mode). The G mode refers to the main vibrational stretching of the graphene in two-dimensional hexagonal lattices, whereas D mode is correlated with the existence of structural defects. These two essential modes of the SWCNT were also detected in the Raman spectra of all coated conjugates and the data are summarized in Table 2. In addition, another important peak, the radial breathing mode (RBM), was seen in the range of 100–300 cm^−1^ for all nanotubes samples, indicating that the structure of the nanotubes has not changed after drug loading and coating process. This important phonon mode in Raman spectra provides information on the nanotubes geometrical parameters and its frequency is inversely proportional to the diameter of a CNT [42]. It is interesting to note that only CS-SWLD conjugate presented the highest Raman intensity with a sharp narrow G mode of all the studied samples. This could be correlated with the changes in orientation effect due to the crystalline supramolecular structure of the CS polymer during the coating treatment [43].

On the other hand, the increase in the intensity ratio between D and G mode (I_D_/I_G_) of the carboxylated SWCNT reflects the relative degree of functionalization or the extent of structural defects present in the nanotubes [44]. The I_D_/I_G_ for all the coated conjugates showed an enhanced value compared to that of SWLD, indicating that the biopolymers generated large cavity after coating treatment, which led to high defect density in SWLD (Table 2). However, this is not the case for CS-SWLD conjugate, in which the intensity ratio of I_D_/I_G_ value decreased significantly from 0.292 to 0.136. This result implies that CS provides the best coverage on SWLD as a protective coating layer. 

**Figure 3:**
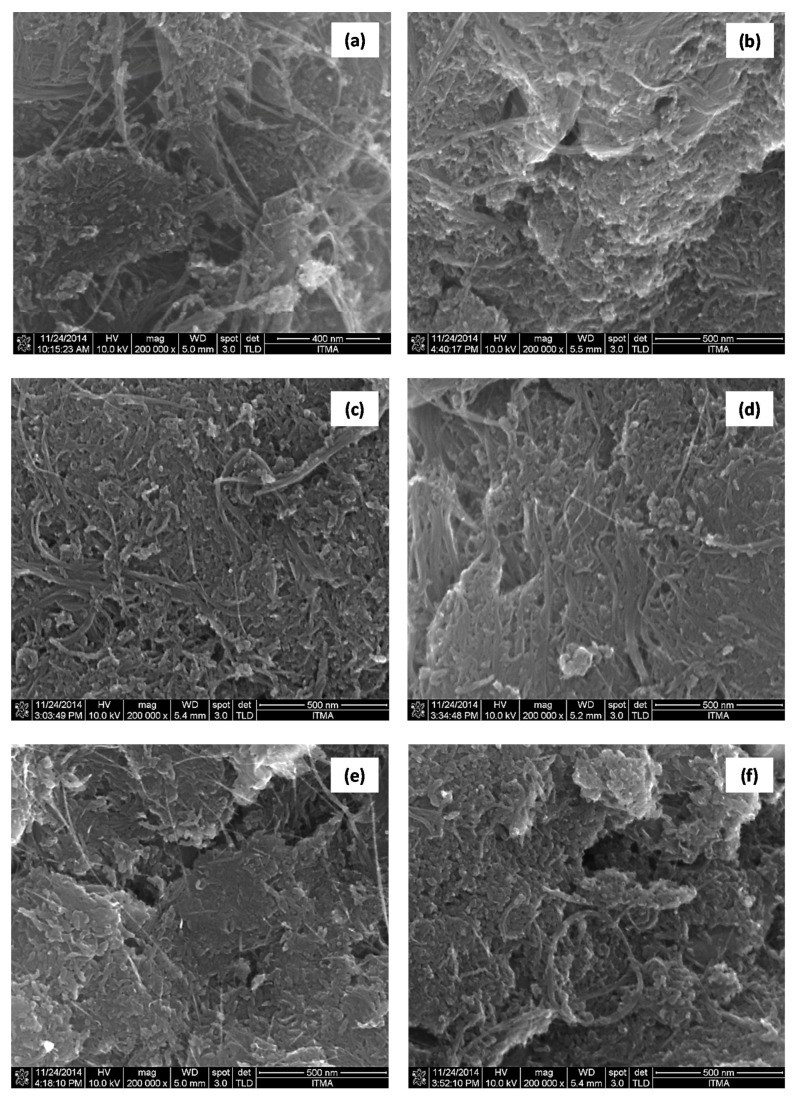
FESEM surface morphologies of: (**a**) carboxylated SWCNT; (**b**) SWLD; (**c**) T2-SWLD; (**d**) T8-SWLD; (**e**) CS-SWLD; and (**f**) PG-SWLD conjugates at magnification of 200,000×.

Figure 3 presents the FESEM surface morphologies of the starting material (carboxylated SWCNT), SWLD and biopolymer-coated SWLD conjugates. It is worth noting that the characteristic tubular feature of the nanotubes is well preserved under all these various surface functionalization steps (drug loading and coating treatment). In particular, CS-SWLD (Figure 3e) was seen to reveal a denser and more compact surface morphology when compared to the others and this explained satisfactorily the decrease of I_D_/I_G_ value, an indicator of defect density, obtained in Table 2.

### 3.2. In Vitro Drug Release of LD

**Figure 4:**
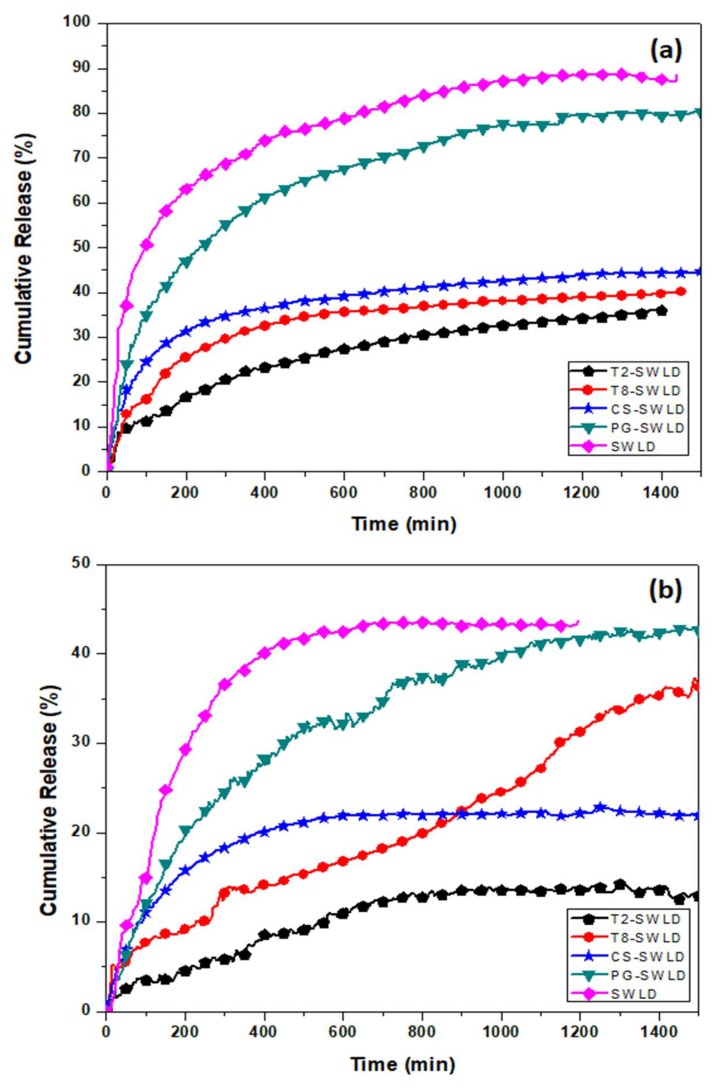
The cumulative release rate of LD from various types of biopolymer coating in PBS solutions at: (**a**) neutral pH 7.4; and (**b**) acidic pH 4.8.

The pH of the release media is a crucial factor for studying drug release process in nanoparticles. Therefore, we investigated the effect of LD in different pH levels, particularly at pH 7.4 and pH 4.8, based on a real-time cumulative drug release experiment. A slightly alkaline pH value of 7.4 was chosen to simulate the physiological environment of human body, whereas pH 4.8 is to mimic the condition of human stomach after food consumption. Based on Figure 4a, initial drug release (or better known as burst release) for biopolymer coated samples was observed to reduce considerably from 32% (uncoated SWLD) to approximately 7–15% in the first 30 min. Burst release is a phenomenon in which an initial large bolus of drug is released immediately upon placement in the release media before reaching stable condition. The burst effect is useful in certain medical applications such as wound treatment to promote immediate pain relief. However, it is not favorable in this context because it can lead to negative consequences such as local or systemic toxicity due to initial high drug concentrations and short half-life of drugs in vivo [45]. Therefore, an effective coating system such as surfactant (e.g., Tweens) or biocompatible polymer (e.g., CS and PG) is desired to protect the drug from fast-release and, at the same time, allow sustained release to prolong the therapeutic action of drug in the circulation half-life. In addition, this may greatly benefit the treatment for PD patients being that LD’s elimination half-life is relatively short, about 50 min, without carbidopa [46].

At pH 7.4, LD was released at a slow and sustained manner from SWLD, T2-SWLD, T8-SWLD, CS-SWLD and PG-SWLD, to the extent of around 89%, 36%, 40%, 45% and 80%, respectively, after 1500 min (Figure 4a). On the other hand, a faster but lower amount of LD was released at pH 4.8 from SWLD (~44%), T2-SWLD (~13%), T8-SWLD (~36%), CS-SWLD (~22%) and PG-SWLD (~43%) until a plateau was reached at approximately 600 min (Figure 4b). The rapid release of LD into pH 4.8 at the initial stage could be affected by the acidity of the medium which leads to partial dissolution of the polymers. Overall, the release rate of LD at pH 7.4 was found to be remarkably higher than that at pH 4.8, suggesting that the release mechanism of LD is pH-triggered. In the neutral environment (pH 7.4), the hydrophilic groups of –COOH will facilitate the release of LD as the polymer swelled in the medium. However, the hydrophilic –COOH changed to hydrophobic when exposed to acidic environment (pH 4.8), and tended to form aggregates, possibly by electrostatic interactions [47], hindering the release of LD into the acidic medium.

As an ideal carrier for targeted drug delivery, the administration, absorption and transportation of CNT must be carefully investigated to achieve optimum therapeutic effects. The commonly studied routes of CNT administration include oral [48,49] and injection (subcutaneous injection, abdominal injection and intravenous injection) [50,51]. The pH study (Figure 4) shows that LD is easily released at pH 7.4 which is the blood pH, suggesting that the intravenous delivery is a possible way of administration. According to the literature, well-functionalized CNT are not retained in the mice reticuloendothelial system (e.g., liver and spleen) upon intravenous injection and they are gradually excreted via fecal and renal pathways [52]. As compared to injection, oral is a preferable mode of administration as it is a non-invasive route and widely accepted by patients. However, the greatest challenge exhibited by orally administered LD is its low bioavailability (~30%) which is caused by the metabolism of decarboxylase enzyme in the gut wall [53]. The LD release experiment in this study showed that, generally, the polymer-coated drug conjugates have a higher release at pH 7.4 compared to pH 4.8, indicating that LD will not be released in the stomach but release in the small intestines. In addition, some CNT might also be readily absorbed into the blood stream, thereby further improve the bioavailability of LD and leads to reduction in the frequency of LD administration.

In general, it was found that the drug release characteristic of LD from PG-SWLD exhibited the highest release rate. This could be mainly associated with the polymer’s hydrophilic property which further improves the dispersibility of the hydrophobic nanotubes in the release medium [54]. For the case of Tween-coated conjugates, there were significant differences between the release pattern of LD, in which T8-SWLD demonstrated a higher release rate compared to T2-SWLD at both pH levels. This observation is in line with the chemical structure characterized by T2 and T8 based on their hydrophilic–lipophilic balance (HLB) values. T8 is the most lipophilic in nature (HLB = 15.0) when compared to T2 (HLB = 16.7) and, hence, the former has a higher affinity for lipophilic drugs such as LD. Consequently, more LD molecules are available for diffusion into the outermost layer of the water-filled surface coating and caused a constant slow release into the PBS medium. In addition, the difference in the release rate can be attributed to the various degree of swelling triggered by the repulsion forces among the ionized –COO– groups in the polymeric chain of the two Tween surfactants [55]. To understand further the difference in the drug release pattern of LD from the surface-coated conjugates, we performed a detailed kinetic analysis of the considered drug from the nanoparticle formulation as the dissolution kinetics governs the bioavailability of the drug.

**Figure 5:**
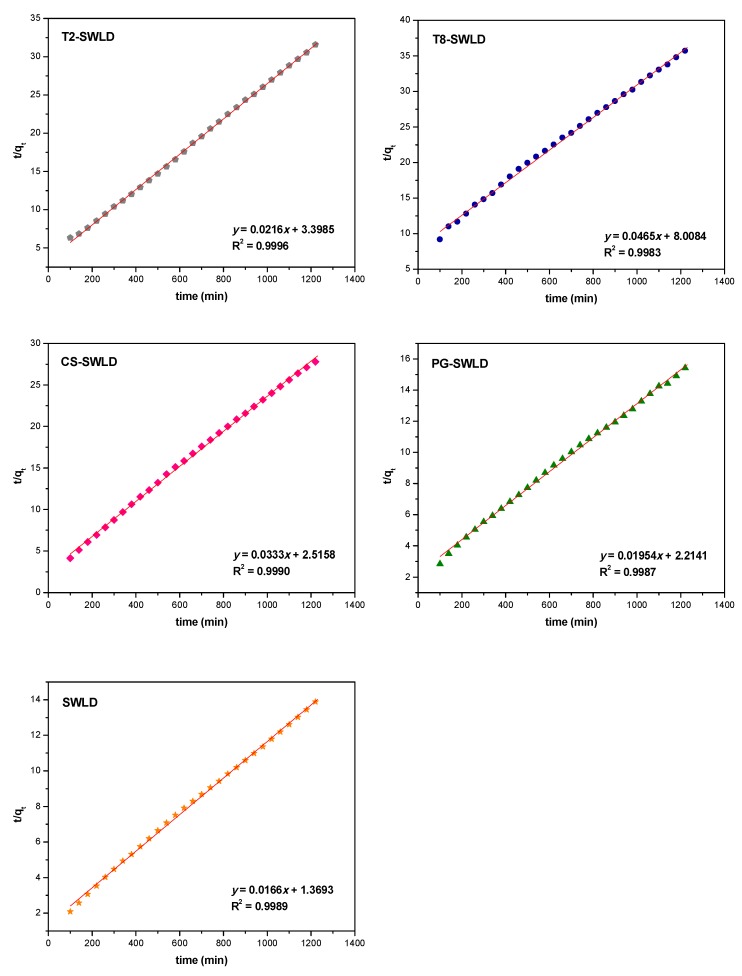
Fits of LD release data from various types of carbon nanotubes conjugate to the pseudo-second order kinetics for pH 7.4 PBS solution.

Five mathematical kinetic models were compared with the aid of equations presented in Table 3 and the correlation coefficient values (R^2^) obtained using these models are also summarized in Table 3. Based on the release kinetics data and the R^2^ values, the release profiles of LD were found to govern by the pseudo-second order (R^2^ = 0.9983–0.9996). This could be attributed to the dissolution kinetics of ions exchanged between the surface-coated SWLD and the anions in the release medium [56]. The model is usually characterized by its linear form, as demonstrated in Figure 5. The rate-controlling step in the drug release process could be due to chemisorption, where the exchange of electrons occurs between the polymer and LD through diffusion mechanism. This is in agreement with previous works on kinetic studies, where the diffusion process is the responsible mechanism that governed the pseudo-second order kinetic [57,58]. On the contrary, when the release medium was changed to pH 4.8, the release profiles of LD from the coated conjugates could not be generalized to a specific equation as they follow different kinetics order for different materials. Thus, it is impossible to elucidate the release profiles of LD in acidic pH compared to neutral pH.

### 3.3. In Vitro Cytotoxicity Assay

**Figure 6:**
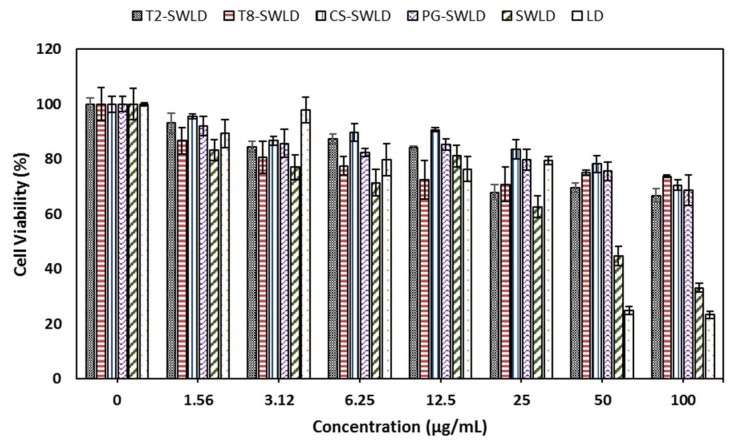
Cytotoxicity effect of T2-SWLD, T8-SWLD, CS-SWLD, PG-SWLD, SWLD and LD on 3T3 cell viability determined by MTT assay after 72 h of incubation.

Mouse embryonic 3T3 fibroblasts were chosen in this initial screening experiment because they represent a non-cancerous (normal) and sensitive cell line. These cells are most commonly used in basal cytotoxicity testing as they are inexpensive, standardized and relatively easy to use [59,60]. The cytotoxicity effect of T2-SWLD, T8-SWLD, CS-SWLD, PG-SWLD, SWLD and LD as determined by MTT assay is demonstrated in Figure 6 and the statistical analysis of the MTT data is summarized in Table 4. As shown in Figure 6, the viability of 3T3 cells treated with increasing concentrations of LD (from 1.56 to 100 μg/mL) resulted in a concentration-dependent decrease as compared to the control. This observation is in accordance with the MTT result published by Kura et al., in which the cell viability of 3T3 cells exposed to pure LD after 72 h of incubation was reduced by more than 50% when the concentration exceeded 100 μg/mL [61]. Therefore, based on our findings, we conclude that higher concentration of LD (>50 μg/mL) may contribute to inhibition of cell proliferation and then led to apoptosis. In addition, SWLD alone showed a cytotoxicity effect in a concentration-dependent manner which demonstrated approximately 50% reduction when concentration exceeded 50 μg/mL. The decrease in cell viability was attributed to the effect of van der Waals forces among the nanotubes and the nanotubes agglomeration may induce a great potential effect in cytotoxicity. Moreover, different production methods may generate changes to their surface characteristics because CNT are known to interact with hydrophobic organic compounds.

To mask the cytotoxicity effect induced by both LD and SWCNT, we subsequently coated SWLD with T2, T8, CS and PG. All four coated formulations did not affect the viability of 3T3 cells in the tested concentration range and maintained greater than 70% cell viability even at 100 μg/mL. These preliminary results indicated that the coating treatment has significantly improved the system’s biocompatibility, and, thus, they are safe to be used for further experiments. Nevertheless, for this new class of nanomaterials, the viability assay of selection has to be ascertained because the interaction between MTT-formazan and SWCNT are highly possible to occur, as reported in the literature [62]. MTT is metabolically reduced to water-insoluble purple formazan crystals by the mitochondrial dehydrogenase and these tetrazolium crystals are then clumped with CNT in the reaction. Consequently, a reduction of MTT-formazan content is detected due to loss of crystals attached to SWCNT and thereby, introducing a fake cytotoxic effect within the assay. As such, to accurately assess the cytotoxicity of these conjugates further investigations are necessary and critical using several well established independent test methods. Among them are lactate dehydrogenase, water-soluble tetrazolium salts (WST) (2,3-bis-(2-methoxy-4-nitro-5-sulfophenyl)-2*H*-tetrazolium-5-carboxanilide) (XTT) and iodonitrotetrazolium chloride assay (INT), and these assessments are currently underway.

## 4. Conclusions

In brief, we report the design and synthesis of a controlled and sustained-release nanodelivery platform based on carboxylated CNT in the presence of different coating agents for the delivery of LD. FTIR analysis showed the presence of functional groups of the coating agents and the drug conjugates, suggesting that the coating process has taken place. All Raman spectra demonstrated the presence of RBM and the two characteristic peaks of CNT which are the D and G modes. This indicates that the structure of the CNT has not changed after drug loading and coating process. The qualitative calculation of I_D_/I_G_ for all coated conjugates showed an increased value after the coating treatment (except for CS-SWLD), suggesting that the biopolymers have generated large cavity and resulted in a considerably high amount of defect density in the drug conjugates. This phenomenon is further supported by the FESEM surface morphology analysis. In vitro drug release experiments conducted in human body-simulated environment at pH 7.4 implied that the drug release profiles of LD were conformed to the pseudo-second order kinetic model. However, the drug release profiles of LD performed at pH 4.8 could not be generalized to a specific model. The cumulative release of LD was observed to exhibit higher release rate at pH 7.4 compared to pH 4.8, indicating that the release rate is pH-triggered. Cytotoxicity was detected in a concentration-dependent manner when pure LD and SWLD were exposed to normal fibroblast cells which showed more than 50% reduction in cell viability at concentration exceeding 50 μg/mL. In contrast, there was no cytotoxicity detected in the cell viability of these fibroblasts incubated with T2-SWLD, T8-SWLD, CS-SWLD and PG-SWLD with similar range of concentration. With the addition of surface coating agents, these conjugates suggest the possibility of a decreased dosing frequency without excessive exposure to large amounts of LD and at the same time, prolonging LD’s efficacy due to the sustained-release ability of the formulation. This is greatly beneficial for treating PD patients and thus further increases patients’ compliance. Nonetheless, there is a need to verify cytotoxicity data with at least two or more independent test systems using water soluble dyes to replace MTT assay.

## Figures and Tables

**Table 1:** Characteristics of the commercial SWCNT provided by Chengdu Organic Chemicals Co. Ltd.

Type	OD (nm)	Length (μm)	Functional Group Content (wt %)	Production Method
**Short SWCNT**	1–2	1–3	2.73	Chemical vapor deposition

**Table 2:** The wavenumber and relative intensity of the D and G mode of all the samples.

Samples	D Mode (cm^−1^)	G Mode (cm^−1^)	I_D_/I_G_
SWCNT (without coating and LD)	1346	1579	0.279
SWLD (without coating)	1350	1579	0.292
T2-SWLD	1342	1583	0.520
T8-SWLD	1350	1591	0.804
CS-SWLD	1342	1583	0.136
PG-SWLD	1350	1587	0.863

**Table 3:** Correlation coefficients obtained by fitting the release of LD from biocompatible polymer-coated conjugates into PBS solutions at pH 7.4 and 4.8.

Formulation	Correlation Coefficient, R^2^				
	Zeroth order (q_t_ = q_0_ + k_0_t)	Pseudo-first order (ln(q_e_ − q_t_) = ln q_e_ − k_1_t)	Pseudo-second order ($\frac{t}{q_{t}}=\frac{1}{k_{2}q_{e}^{2}}+\frac{t}{q_{e}}$)	Korsmeyer–Peppas model ($\frac{q_{t}}{q_{\infty }}=kt^{n})$	Higuchi model ($q_{t}=k_{H}\sqrt{t}$)
PBS solution at pH 7.4					
SWLD	0.8980	0.9503	0.9989	0.9868	0.9651
T2-SWLD	0.7748	0.9847	0.9996	0.9065	0.8762
T8-SWLD	0.9255	0.9917	0.9983	0.9844	0.9811
CS-SWLD	0.8777	0.9735	0.9990	0.9755	0.9485
PG-SWLD	0.8960	0.9899	0.9987	0.9812	0.9637
PBS solution at pH 4.8					
SWLD	0.5839	0.9944	0.9953	0.7854	0.7134
T2-SWLD	0.9071	0.9552	0.9123	0.9650	0.9487
T8-SWLD	0.9802	0.9480	0.8508	0.9656	0.9427
CS-SWLD	0.6551	0.9392	0.9971	0.8516	0.7756
PG-SWLD	0.9249	0.9818	0.9953	0.9786	0.9761

Note: q_t_, q_e_ and q_∞_ represent the released amount of drug in time t, at equilibrium and at infinite time, respectively. k_0_, k_1_, k_2_ and k_H_ indicate the equilibrium rate constants of the models.

**Table 4:** In vitro 3T3 cell viability of T2-SWLD, T8-SWLD, CS-SWLD, PG-SWLD, SWLD and LD obtained by MTT assay (mean ± SD, *n* = 3). Different alphabets indicate statistically significant differences of the cell viability made between the concentrations of the same compound (ANOVA: Duncan’s test, *p* < 0.05).

Concentration (μg/mL)	Cell Viability (%)					
	T2-SWLD	T8-SWLD	CS-SWLD	PG-SWLD	SWLD	LD
1.56	93.1 ± 3.6 ^a^	86.7 ± 4.9 ^a^	95.6 ± 0.8 ^a^	92.1 ± 3.5 ^a^	83.3 ± 3.8 ^a^	89.4 ± 5.1 ^b^
3.12	84.5 ± 1.9 ^c^	80.7 ± 5.9 ^b^	86.7 ± 1.6 ^b,c^	85.7 ± 5.1 ^b^	77.1 ± 4.5 ^a,b^	97.8 ± 4.7 ^a^
6.25	87.4 ± 1.7 ^b^	77.5 ± 3.3 ^b,c^	89.6 ± 3.2 ^b^	82.4 ± 1.4 ^b,c^	71.5 ± 4.8 ^b,c^	79.8 ± 5.8 ^c^
12.50	84.1 ± 0.8 ^c^	72.5 ± 6.9 ^c,d^	90.7 ± 0.8 ^a,b^	85.2 ± 2.0 ^b^	81.1 ± 3.9 ^a^	76.4 ± 4.5 ^c^
25.00	67.9 ± 2.7 ^d,e^	70.8 ± 6.3 ^d^	83.6 ± 3.5 ^c^	79.9 ±3.7 ^c^	62.7 ± 3.9 ^c^	79.5 ± 1.5 ^c^
50.00	69.5 ± 1.8 ^d^	75.1 ± 0.9 ^c,d^	78.3 ± 3.0 ^d^	75.6 ± 3.3 ^d^	44.6 ± 3.5 ^d^	24.9 ± 1.6 ^d^
100.00	66.8 ± 2.6 ^e^	73.7 ± 0.5 ^c,d^	70.5 ± 1.9 ^e^	68.6 ± 5.5 ^e^	33.1 ± 1.6 ^e^	23.3 ± 1.3 ^d^
